# Comparative Transcriptome Analysis of Chemoreception Organs of *Laodelphax striatellus* in Response to Rice Stripe Virus Infection

**DOI:** 10.3390/ijms221910299

**Published:** 2021-09-24

**Authors:** Yao Li, Yunye Zhang, Yin Xiang, Danyu Chen, Jia Hu, Fang Liu

**Affiliations:** 1 College of Horticulture and Plant Protection, Yangzhou University, Yangzhou 225000, China; liyao@yzu.edu.cn (Y.L.); 13451634325@163.com (Y.Z.); xiangying9262021@163.com (Y.X.); 17715120593@163.com (D.C.); hj199412311231@163.com (J.H.); 2 State Key Laboratory for Biology of Plant Diseases and Insect Pests, Institute of Plant Protection, Chinese Academy of Agricultural Sciences, Beijing 100081, China; 3 Jiangsu Co-Innovation Center for Modern Production Technology of Grain Crops, Agricultural College, Yangzhou University, Yangzhou 225000, China; 4 Joint International Research Laboratory of Agriculture and Agri-Product Safety, The Ministry of Education of China, Institutes of Agricultural Science and Technology Development, Yangzhou University, Yangzhou 225000, China

**Keywords:** rice stripe virus, *Laodelphax striatellus*, chemoreception organs, olfactory related genes

## Abstract

Many vector-borne viruses possess the ability to manipulate vector behaviors to facilitate their transmission. There is evidence that the mechanism of this phenomenon has been described in part as direct manipulation through regulating vector chemosensation. Rice stripe virus (RSV) is transmitted by the small brown planthopper, *Laodelphax striatellus* (Fallen), in a persistent, circulative–propagative manner. The effect of RSV infection on the olfactory system of *L. striatellus* has not been fully elucidated. Here, we employed transcriptomic sequencing to analyze gene expression profiles in antennae, legs and heads (without antennae) from *L. striatellus* females and males with/without RSV infection. Comparisons of the differentially expressed genes (DEGs) among antennae, legs and heads indicated that tissue-specific changes in the gene expression profile were greater than sex-specific changes. A total of 17 olfactory related genes were differentially expressed in viruliferous antennae as compared to nonviruliferous antennae, including *LstrOBP4/9, LstrCSP1/2/5*, *LstrGR28a/43a/43a-1*, *LstrIR1/2/NMDA1*, *LstrOR67/85e/56a/94* and *LstrSNMP2/2-2*. There are 23 olfactory related DEGs between viruliferous and nonviruliferous legs, including *LstrOBP2/3/4/12/13*, *LstrCSP13/5/10*, *LstrIR1/2/Delta2/Delta2-1/kainate2/NMDA2*, *LstrOR12/21/31/68* and *LstrORco*. A low number of olfactory related DEGs were found between viruliferous and nonviruliferous heads, including *LstrCSP1*, *LstrOBP2*, *LstrOR67* and *LstrSNMP2-2*. Among these DEGs, the expression patterns of *LstrOBP2*, *LstrOBP3* and *LstrOBP9* in three tissues was validated by quantitative real-time PCR. The demonstration of overall changes in the genes in *L. striatellus’* chemoreception organs in response to RSV infection would not only improve our understanding of the effect of RSV on the olfactory related genes of insect vectors but also provide insights into developing approaches to control the plant virus transmission and spread as well as pest management in the future.

## 1. Introduction

Vector-borne viruses contribute to a substantial portion of the global plant disease burden, accounting for more than 70% of all known plant viruses [1,2]. The horizontal transmission and spread of vector-borne viruses relies on the movement of insect vectors, which could be manipulated by vector-borne viruses [3,4]. The mechanism by which viruses manipulate vector behaviors has largely been described as indirect manipulation through modifications of the quality, color, odor and associated plant traits. For instance, cucumber mosaic virus (CMV) accelerates the emigration of aphid vectors by reducing host plant quality, and increased the attractiveness of infected plants to aphid vectors by inducing elevated emissions of a volatile blend [3]. Potato leafroll virus (PLV) infection changes the volatile blend of potato to attract vector aphids, thereby increasing the probability of virus acquisition [5]. Furthermore, some vector-borne viruses could manipulate vector behavior via directly affecting the nervous and olfactory system. Tomato yellow leaf curl virus (TYLCV) reduces whitefly preference to viruliferous plant by disabling the nervous system of vectors [6]. Southern rice black streaked dwarf virus (SRBDV) and tomato chlorosis virus (TCV) are able to regulate the gene expression of *odorant-binding protein* (*OBP*) 2 and *OBP*3 in their vectors, *Sogatella furcifera* and *Bemisia tabaci*, respectively, and to reverse the preferences of nonviruliferous/viruliferous vectors, finally affecting the virus transmission [7,8]. Therefore, various vector-borne viruses have developed the ability to utilize a direct or indirect manipulation mechanism to induce behavioral changes of insect vectors, to benefit their spread. However, the effect of vector-borne virus infection on the olfactory system of insect vectors has not been fully elucidated.

The critical roles of the insect’s olfactory system in their locomotion, host seeking, probing and feeding are mainly executed via detecting semiochemicals by sensilla on the main periphery, such as the main chemoreception organ, the antennae [9,10]. A number of protein families located in these peripheral sensory organs are responsible for the recognition of semiochemicals, which include two classes of carrier proteins: OBPs and chemosensory proteins (CSPs), and four classes of membrane proteins: ionotropic (IR), gustatory (GRs), membrane-bound odorant receptors (ORs)/olfactory co-receptor receptor (ORco) and sensory neuron membrane proteins (SNMPs) [11,12,13,14,15,16]. When environmental chemicals penetrate the pores of sensilla, OBPs and CSPs as carriers bind these chemicals in the sensilla lymph and transport the chemicals to ORs or IRs located on the olfactory sensory neurons. Then, chemical signals are converted into electrical signals, and conveyed to the insect’s brain to eventually modulate the insect’s behavior [14,17]. Thus far, the role of olfactory-related proteins in virus transmission remains unclear.

Rice stripe virus (RSV), a typical member of the genus *Tenuivirus*, has inflicted serious yield losses of rice by causing the notorious rice stripe disease in Eastern Asia [18,19]. It is transmitted by the small brown planthopper, *Laodelphax striatellus* (Fallen), in a persistent, circulative–propagative manner [20,21,22]. RSV particles enter *L. striatellus* via the alimentary canal during feeding and initially establish infection in the midgut epithelium, propagating in midgut visceral muscles. After transferring into the hemolymph, these virus invade several key organs via lymph circulation and, ultimately, arrive at the salivary gland, from which RSV particles are inoculated back to the plant hosts along with saliva. RSV transmission in *L. striatellus* requires specialized interactions between components of the virus and vector, evidenced by the transcriptome analyses of the expression changes of overall mRNA genes and the regulation of RSV-derived siRNA in *L. striatellus* after RSV infection [23,24,25]. In addition, the organ-specific transcriptomes of *L. striatellus’* alimentary canal and salivary gland characterize the responses of two organs in confronting RSV infection [26]. However, the molecular mechanism underlying the interactions between RSV and chemoreception organs remains unknown at the omic level.

Our preliminary antennae-specific transcriptome analysis identified 14 *OBPs*, 12 *CSPs*, 7 *SNMPs* and *95 ORs* in *L. striatellus* [27]. Meanwhile, viruliferous *L. striatellus* might have a stronger olfactory and seeking ability for rice than nonviruliferous insect [28]. Thus, the current study aims to further investigate the host preference mechanism of viruliferous *L. striatellus* and the role of the olfactory system in RSV transmission. We compared the response of the antennae, legs and heads (without antennae) from both *L. striatellus* females and males to RSV infection using next-generation deep-sequencing techniques. Furthermore, olfactory related genes from differentially expressed genes (DEGs) of chemoreception organ transcriptomes were enriched between viruliferous and nonviruliferous organs. Lastly, a qPCR was performed to confirm the expression changes of three OBPs between viruliferous and nonviruliferous tissues. Our results would not only improve our understanding on the role of the olfactory chemoreception organs system in RSV infection and transmission but also provide new insights into the symbiosis interaction between virus, insect and plant. A deeper understanding of this interaction provides new avenues for controlling plant viruses and their vectors.

## 2. Results

### 2.1. Illumina Sequencing and Assembly of the Chemoreception Organs of Female and Male Laodelphax striatellus

Twelve cDNA libraries were obtained from dissected antennae, heads and legs of male and female *L. striatellus* with or without RSV. The transcriptomes of twelve libraries (Viruliferous female antennae, VFA; Viruliferous male antennae, VMA; Viruliferous female legs, VFL; Viruliferous male legs, VML; Viruliferous female heads, VFH; Viruliferous male heads, VMH; Nonviruliferous female antennae, NFA; Nonviruliferous male antennae, NMA; Nonviruliferous female legs, NFL; Nonviruliferous male legs, NML; Nonviruliferous female heads, NFH; Nonviruliferous male heads, NMH) were sequenced using the Illumina HiSeq™ 2000 platform, and generated 663,707,248 clean sequence reads, containing 99.55 G bp clean bases (Table S1). GC percentages (%) of sequence reads from the twelve libraries were all approximately 40%, and Q20 (%) were more than 95.88%, which showed that the accuracy and quality of the sequencing data met the standard for further analysis (Table S1). Of the reads from each transcriptome, 58.06~68.94% paired-end clean reads were mapped to the genome of *L. striatellus* (Table S1) and assembled into 20,409 genes with function annotation. Additionally, the normalized read count with Fragments per Kilobase of transcript per Million mapped reads (FPKM) of these genes were calculated.

**Figure 1:**
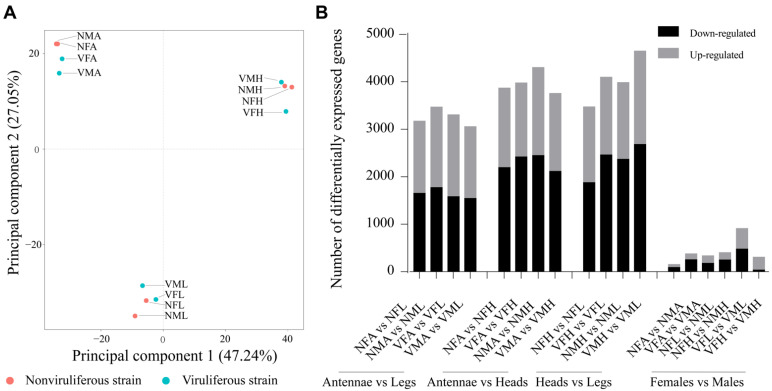
The transcript abundance in antennae, legs and heads of female and male *L. striatellus.* (**A**) Principal component analysis of the transcriptomes from the three organs of viruliferous/nonviruliferous *L. striatellus* females and males. Viruliferous transcriptomes are denoted by green dots, and nonviruliferous transcriptomes are denoted by pink dots. (**B**) The total number of DEGs from comparisons among three organs or between females and males. The number of down-regulated DEGs is denoted by black, and the number of up-regulated DEGs shown by gray.

Principal component analysis (PCA) was used to compare the twelve transcriptomes from viruliferous and nonviruliferous organs of females and males. Overall, the principal component axis 1 and 2 (PC1 and PC2, accounting for 47.24% and 27.05% of the observed gene expression variation, respectively), separated these libraries into three groups: Antennae libraries (VMA, VFA, NMA and NFA), Legs libraries (VML, VFL, NML and NFL) and Heads libraries (VMH, VFH, NMH and NFH) (Figure 1A). The comparison between the three tissues demonstrated the relative most number of DEGs between heads and legs (especially in VMH versus (vs.) VML), modest in antennae vs. heads, and least in antennae vs. legs. However, the comparison between female tissues and male tissues showed fewer DEGs (Figure 1B). These results demonstrated that organ-specific and sex-specific DEGs profile in *L. striatellus* might be involved in the host preference of viruliferous *L. striatellus*.

### 2.2. Gene Ontology Classification of Differentially Expressed Genes among the Chemoreception Organs of Females and Males

**Figure 2:**
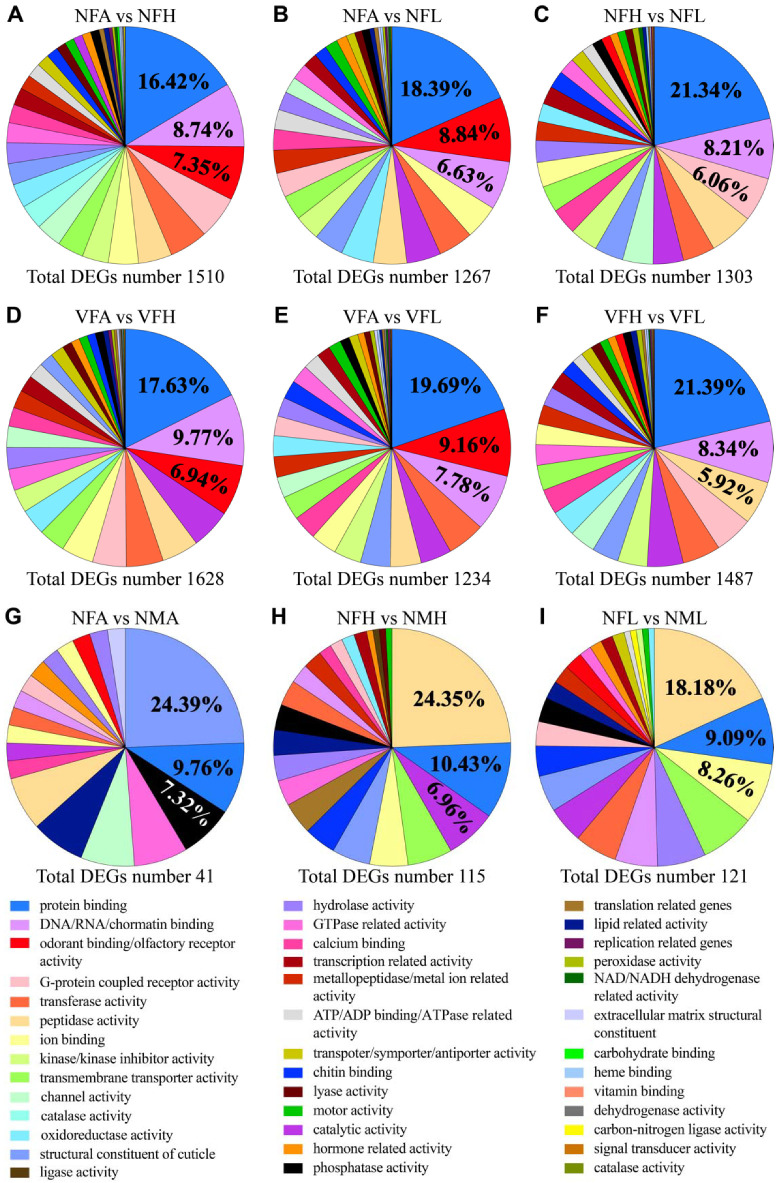
Proportions of DEGs among (**A**–**F**) three organs or (**G**–**I**) between two sexes classified by a level 1 molecular function gene ontology. The legend indicates the top three listed GO (Gene Ontology) terms in at least one pairwise comparison.

The DEGs among the antennae, legs and heads were functionally annotated and classified into different molecular function categories by gene ontology (GO) analysis (Figure 2). Eight pairwise comparisons between antennae and other tissues revealed a total of 1628, 1234, 1510, 1267, 1451, 1107, 1514 and 1409 DEGs with GO analysis. There were approximately 37 significantly enriched GO categories concerning molecular function seen in the DEGs of eight comparisons, of which the top three significant GO terms were protein binding (16.42~21.77%), DNA/RNA/chromatin binding (6.63~9.77%) and odorant-binding/olfactory receptor activity (6.94~10.12%) (Figure 2A,B,D,E and Figure S1A,B,D,E). In the DEGs’ molecular function of heads vs. legs, approximately 35 significantly enriched GO categories were identified with the top three GO terms involved in protein binding, DNA/RNA/chromatin binding and *G*-protein coupled receptor activity or peptidase activity (Figure 2C,F and Figure S1C,F).

When comparing the transcriptomes between females and males, only 152, 93, 286, 41, 115 and 121 genes were differentially expressed between VFA vs. VMA, VFL vs. VML, VFH vs. VMH, NFA vs. NMA, NFL vs. NML, NFH vs. NMH, respectively (Figure 2G–I and Figure S1G–I). The enriched GO terms for these sex-specific DEGs among the three tissues include protein binding, structural constituent of the ribosome, phosphatase activity, peptidase activity, and so on.

Taken together, these results demonstrated that a large number of genes involved in the regulation of cellular environment and peripheral chemosensory were more or less expressed in chemoreception organs, antennae and legs, but a low number of DEGs were found between females and males. It suggested that these organ- and sex-specific DEGs might participate in the host preference of *L. striatellus* on rice, not considering whether the samples are from viruliferous or nonviruliferous *L. striatellus*.

### 2.3. Differentially Expressed Genes Induced by RSV Infection

**Figure 3:**
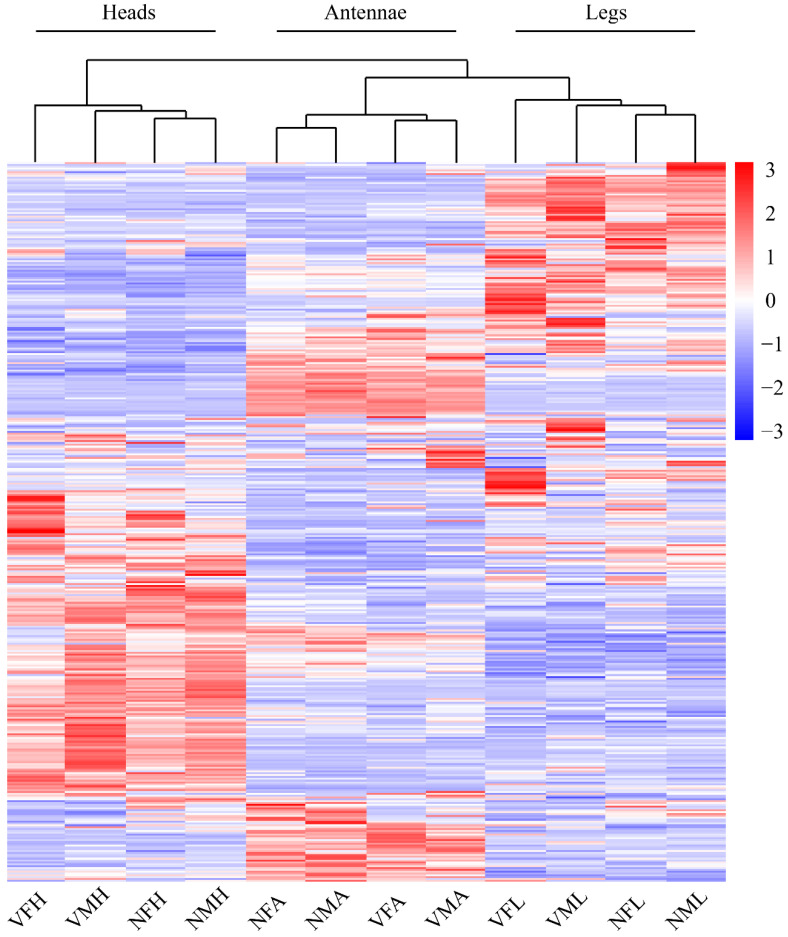
The expression profiles of DEGs between viruliferous and nonviruliferous organs from female and male *L. striatellus*. All DEGs expression were performed in each organ in both males and females *L. striatellus* with or without RSV infection by heatmap. The expression profiles of each gene were calculated based on log_2_FPKM.

**Figure 4:**
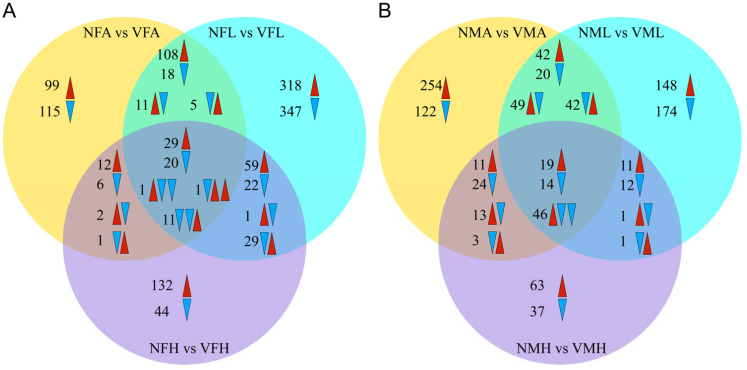
The changes in DEGs expression between viruliferous and nonviruliferous organs from female and male *L. striatellus*. (**A**) Venn diagram summary of DEGs comparing VFA vs. NFA, VFL vs. NFL, VFH vs. NFH. (**B**) Venn diagram summary of DEGs comparing VMA vs. NMA, VML vs. NML, VMH vs. NMH. The up-regulated DEGs are denoted by red arrows, and the down-regulated DEGs shown as blue arrows.

Thus, we next inspected the gene expression profile of viruliferous and nonviruliferous organs through a comparative heatmap. The gene expression patterns for the same organ clustered together. Among these clustered samples, the viruliferous samples were outgrouped from the nonviruliferous samples (Figure 3). According to the Venn diagram, the comparison between viruliferous and nonviruliferous female tissues revealed 439 (262 Up, 177 Down), 980 (521 Up, 459 Down) and 370 (274 Up, 96 Down) DEGs in antennae, legs and heads, respectively. Among them, there were 62 DEGs shared to responses to RSV infection (with 29 up-regulated and 20 down-regulated in all three tissues) (Figure 4A). Similarly, comparisons between viruliferous and nonviruliferous male tissues identified 659, 579 and 255 DEGs, respectively. The three tissues of males shared 79 DEGs in response to RSV infection (Figure 4B).

### 2.4. Gene Ontology Classification of Differentially Expressed Genes Induced by RSV Infection

**Figure 5:**
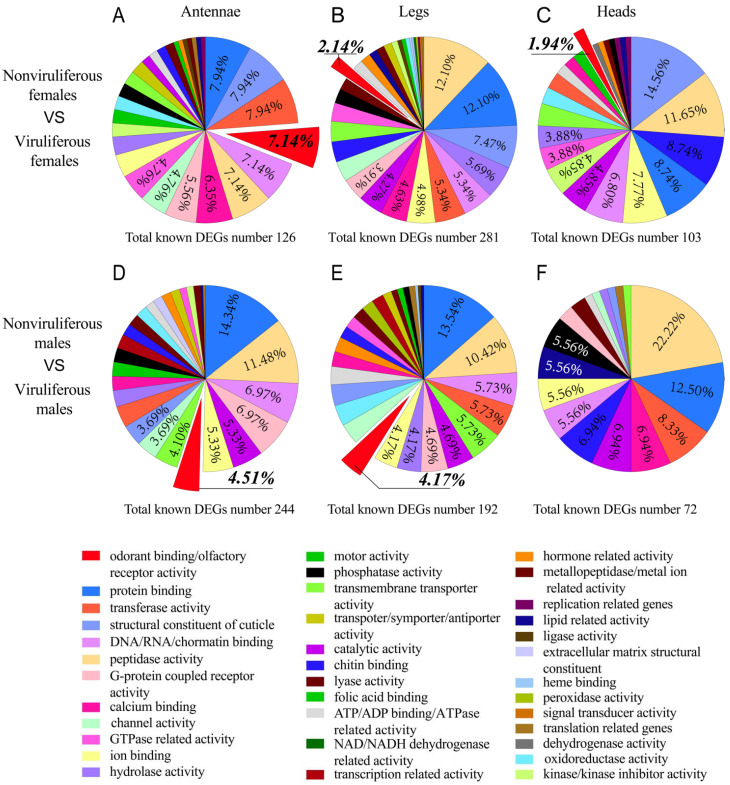
GO analysis of DEGs between viruliferous and nonviruliferous organs. Proportions of DEGs between viruliferous and nonviruliferous (**A**,**D**) antennae/(**B**,**E**) legs/(**C**,**F**) heads classified by a level 1 molecular function gene ontology. The legend indicates the top ten listed GO terms in at least one pairwise comparison.

Hundreds of DEGs between viruliferous and nonviruliferous tissues were functionally annotated by GO analysis and were classified into 28~33 significantly enriched categories between viruliferous and nonviruliferous organs (Figure 5A–F). The top ten GO categories included protein binding, peptidase activity, structural constituent of cuticle, DNA/RNA/nucleotide/chromatin binding, transferase activity, transmembrane transporter activity, hydrolase activity, catalytic activity, ion binding and odorant-binding/olfactory receptor activity (Figure 5A,B,D,E). There were only 18 and 24 significantly enriched GO terms from nonviruliferous vs. viruliferous male and female heads, respectively (Figure 5C,F). In particular, a large number of olfactory-related genes (7.14%, 4.51%, 2.14% and 4.17%) were found to be more or less enriched in viruliferous chemoreception organs (antennae and legs) as compared to nonviruliferous chemoreception organs (Figure 5A,B,D,E). The data indicated a link of the olfactory related genes to the difference between the viruliferous and nonviruliferous chemoreception organs, however, deep analysis of these genes may shed light on the interaction between RSV and the vector’s chemoreception organs.

### 2.5. Changes in the Expression of Olfactory-Related Genes in the Chemoreception Organs Infected by RSV

**Figure 6:**
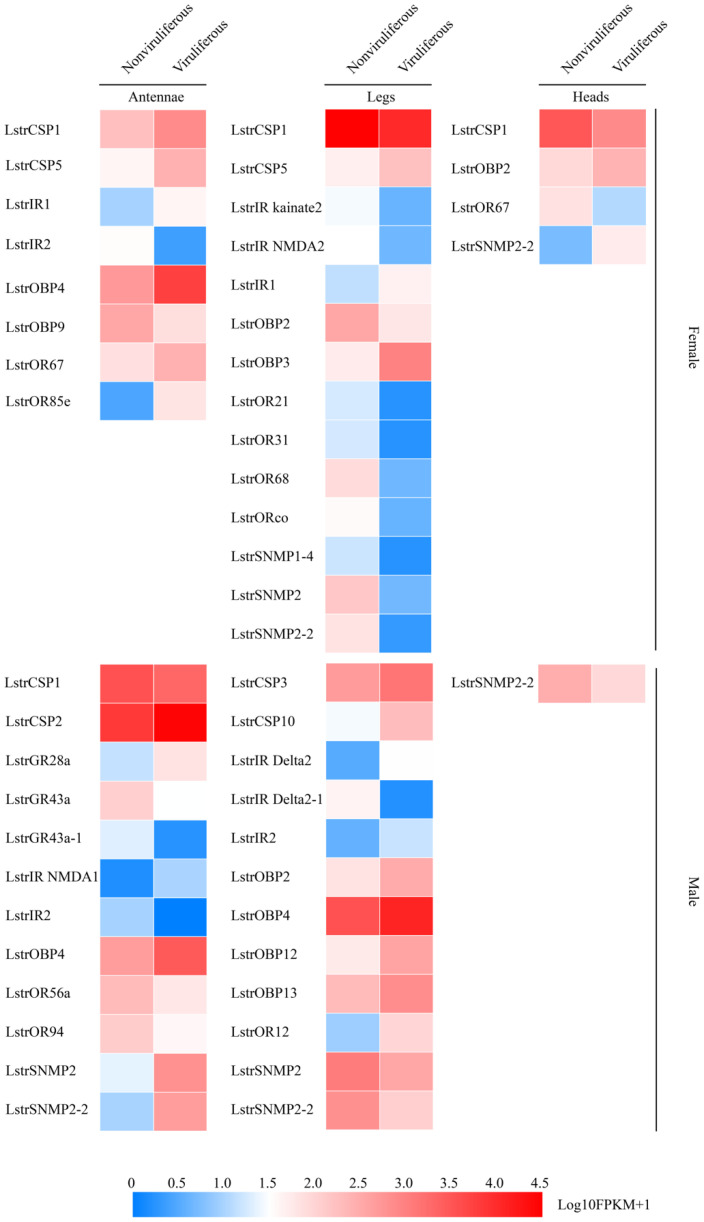
The expression profiles of differentially abundant olfactory-related genes between viruliferous and nonviruliferous female/male organs. The expression profiles of each gene were calculated based on log_10_FPKM+1.

In order to validate the effect of RSV infection on the olfactory system, a more in-depth expression profiling the olfactory-related chemosensation and olfaction DEGs was performed. A total of 17 olfactory-related genes were differentially expressed in viruliferous antennae compared to nonviruliferous antennae (Figure 6 left). A cluster of olfactory genes, including *LstrOBP4*, *LstrCSP2/5*, *LstrGR28a*, *LstrIR1/NMDA1*, *LstrOR67/85e* and *LstrSNMP2/2-2*, showed higher abundance after infection in both male and female antennae (Figure 6 left). The expression level of six other olfactory genes (*LstrOBP9*, *LstrGR43a*/*43a-1*, *LstrIR2* and *LstrOR56a*/*94*) decreased in both male and female viruliferous antennae (Figure 6 left). Between viruliferous and nonviruliferous legs, a total of 23 olfactory-related genes were found to be differentially expressed (down-regulated: *LstrCSP1*, *LstrIR kainate2/NMDA2/Delta2-1*, *LstrOR21/31/68*, *LstrORco* and *LstrSNMP1-4/2/2-2*; up-regulated: *LstrCSP3/5/10*, *LstrIR1/2/Delta2*, *LstrOBP3/4/12/13* and *LstrOR12*) (Figure 6 middle). Comparative gene expression profiling of viruliferous vs. nonviruliferous heads revealed only four DEGs (*LstrCSP1*, *LstrOBP2*, *LstrOR67* and *LstrSNMP2-2*) (Figure 6 right). Interestingly, three olfactory genes of these DEGs (*LstrCSP1*, *LstrOBP2*, *LstrSNMP2-2*) appeared to show opposite changes between female and male organs after RSV infection (Figure 6). Consistently, the influence of RSV infection on the expression of olfactory-related genes in antennae and legs was greater than that in heads (without antennae).

### 2.6. Validation of the OBPs Expressions in RSV-Infected Chemoreception Organs by Quantitative Real-Time PCR

**Figure 7:**
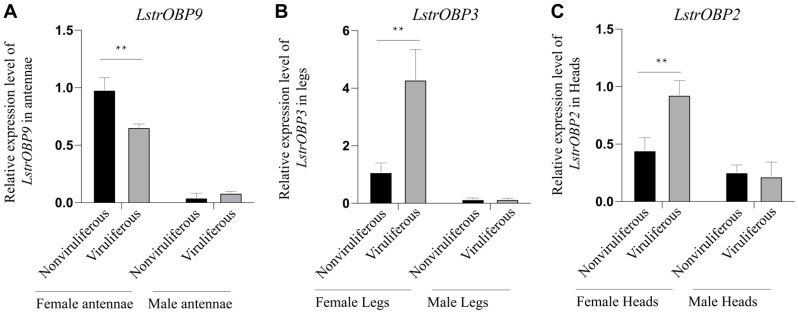
Relative expression pattern of three selected olfactory-related genes in viruliferous/nonviruliferous female and male organs. (**A**) Expression changes of *LstrOBP9* between viruliferous and nonviruliferous antennae. (**B**) Expression changes of *LstrOBP3* between viruliferous and nonviruliferous legs. (**C**) Expression changes of *LstrOBP2* between viruliferous and nonviruliferous heads. ** indicated *p* < 0.01.

To confirm the above transcriptome results, we selected a significant differentially expression OBP from each organ transcriptome for qPCR examination. The expression of selected *LstrOBP9* gene in viruliferous female antennae was significantly down-regulated by 33.1% compared to that in nonviruliferous female antennae, while it exhibited no significant change in viruliferous vs. nonviruliferous male antennae (Figure 7A). On the contrary, the expression level *LstrOBP3* in viruliferous female legs was about 4-fold of that in nonviruliferous female legs (Figure 7B). Similarly, the *LstrOBP2* was also highly expressed in the heads of viruliferous female, but no changes were found between viruliferous and nonviruliferous male heads (Figure 7C). In general, the expression trends of the three *LstrOBPs* were consistent with our transcriptome data, supporting the involvement of these organ- and sex-specific olfactory-related *LstrOBPs* in mediating the host preference of *L. striatellus* on rice plant behavior as well as RSV transmission.

## 3. Discussion

The small brown planthopper, as one of the most destructive rice pests, is notorious for causing serious yield loss by feeding damage as well as transmitting RSV [29]. Before the release of the *L. striatellus* genome sequence in 2017 [30], several previous pyrosequencing-based transcriptome studies have characterized the whole body or organ transcriptomic response [23,26]. In the current study, based on a high-quality genome assembly and annotation of *L. striatellus,* we performed transcriptomic RNA sequencing to analyze gene expression profiles in three tissues (antennae, legs and heads) from *L. striatellus* females and males with/without RSV infection. The identification of these DEGs would provide insights into the host preference of *L. striatellus* on rice plant behavior as well as RSV transmission.

The GO analysis for these DEGs found that the top listed GO terms included protein binding, DNA/RNA/chromatin binding, odorant-binding/olfactory receptor activity, *G*-protein coupled receptor activity and peptidase activity, suggesting a tissue- or sex- specific signaling reaction. Moreover, the comparison of DEGs between antennae vs. legs/heads was more enriched in the regulation of the cell cycle, cellular environment and peripheral chemosensory than those of heads vs. legs. Since most olfactory sensilla of insects are distributed on the surface of primary chemoreception organs, the antennae, while insect legs and heads are secondary chemoreception organs and olfactory signal processing organs, respectively [31], it is reasonable that olfactory-related genes were higher expressed in the antennae than that in the legs and heads. Thus, the increase or decrease in olfactory-related genes in antennae after virus infection has dire implications for vectorial capacity.

To the best of our knowledge, there has been no systematic study on the effect of virus infection on the olfactory genes expression of insect vector’s antennae. Our study demonstrated overall changes in insect chemoreception organs in response to infection with plant virus RSV. Transcriptomic comparisons of viruliferous vs. nonviruliferous chemoreception organs indicated that a large number of olfactory related genes were differentially expressed in viruliferous antennae and legs but not in the heads. A previous report also found that dengue virus could infect the mosquito’s antennae and result in changes in the transcript abundance of two *AgOBPs* [32]. The SRBDV infection led to a decrease in *SfOBP2* and *SfOBP11* in *S. furcifera* [7]. However, the TCV infection increased the expression of *BtOBP3* in *B. tabaci* [8]. Li and his colleagues found that the *LstrORco* was stimulated by RSV in the head (with antennae) [28]. However, our data showed that RSV infection down-regulated the *LstrORco* expression in female *L. striatellus* legs and did not affect the *LstrORco* expression in antennae and heads (without antennae). These different results may be due to different sampling and detection methods. The qPCR method was used to detect the *LstrORco* expression in mixed samples of heads and antennae in Li and his colleagues’ study, while transcriptome sequencing was used to detect the *LstrORco* expression in antennae and heads (without antennae) in our study. The protein level expression and the location of *LstrORco* in chemoreception organs need to be further verified by Western blot and in situ hybridization.

Previous reports have demonstrated that plant viruses could regulate olfactory genes to affect the behavior of insect vectors and their associations with host plants. SRBDV and TCV reversed the preferences of insect vectors by regulating OBPs [7,8]. Li and his colleagues reported that silencing of the *LstrORco* expression inhibited the host seeking behavior and increased the ‘no response’ percent and the response time of *L. striatellus* [28]. Our transcriptome analysis revealed 17 olfactory-related genes differentially expressed in viruliferous antennae (including two *LstrOBPs*, three *LstrCSP*s, three *LstrGR*s, three *LstrIR*s, four *LstrOR*s and two *LstrSNMP*s) as well as five *LstrOBP*s, three *LstrCSP*s, six *LstrIR*s, four *LstrOR*s and *LstrORco* differentially expressed in viruliferous legs. Additionally, only four olfactory genes were differentially abundant in viruliferous heads. During insect chemical perception, OBPs and CSPs can selectively transport specific odorants to four classes of membrane proteins for odorant recognition [14]. It is possible that the expression changes in olfactory-related genes alter the plant’s volatile organic compound (VOC) recognition and affect insect behavioral responses. Taken together, it was implied that the RSV induction of olfactory related genes, including *LstrORco*, in antennae and legs could alter the feeding behavior of *L. striatellus* on rice with the host preference and, thus, at least theoretically, affect virus transmission. In the next step, the plant VOCs recognized by differentially expressed olfactory genes can be screened using gas chromatography–electroantennographic detection (GC–EAD). The two electrodes voltage clamp technique and field trapping experiments can be conducted to validate the interaction between differentially expressed olfactory genes and plant VOCs. Through these techniques, we will further reveal the role of olfactory genes in RSV-induced behavior.

## 4. Materials and Methods

### 4.1. Nonviruliferous and Viruliferous L. striatellus Rearing

Nonviruliferous and viruliferous strains of *L. striatellus* were a gift from Prof. Yijun Zhou’s laboratory of Jiangsu Academy of Agricultural Sciences, Jiangsu province, China, and were reared independently on seedlings of rice cv. Wuyujing 3 in a growth incubator at 25 ± 1 °C, with 80% ± 5% RH and a 12-h light–dark photoperiod.

To ensure that insects were viruliferous, individual female insects were allowed to feed independently. The presence of RSV in their offspring were collected and detected via Dot-ELISA with the monoclonal anti-CP antibody [33]. Highly viruliferous colonies were screened and prepared for subsequent studies.

### 4.2. Samples Preparation and Transcriptomic Sequencing

The antennae, legs and heads (without antennae) of more than 3000 *L. striatellus* adults were dissected independently. Viruliferous female/male and nonviruliferous female/male samples were collected and stored at −80 °C for each RNA library. TRIzol method was used to extract total RNA from organ samples as recommended by the manufacturer (Ambion, Austin, TX, USA). RNA purity and concentration were checked using the NanoPhotometer^®^ spectrophotometer (IMPLEN, Westlake Village, CA, USA) and RNA assay kit in Qubit^®^ 2.0 Fluorometer (Life Technologies, Carlsbad, CA, USA). Twelve paired-end RNA-seq libraries were established from the extracted RNA using the NEBNext^®^ Ultra™ RNA Library Prep Kit (New England BioLabs, Ipswich, MA, USA). First and second strand cDNA were synthesized according to the methods described previously [27]. After the adapters had been attached, PCR was performed with the adapter-ligated cDNA as templates, phusion high-fidelity DNA polymerase, universal PCR primers and index (X) Primer to generate cDNA libraries. These libraries were sequenced with Illumina HiSeq 2000 sequencer (Illumina, San Diego, CA, USA) and 125–150 bp paired-end reads were generated.

### 4.3. Transcriptomic Assembly and GO Annotation

After sequencing, FastQC was employed to check the quality distribution of the raw data (http://www.bioinformatics.babraham.ac.uk/projects/fastqc, accessed on 20 December 2018). The clean reads were obtained by removing reads containing adapter, reads containing ploy-N and low quality reads from raw reads. The Q20, Q30 and GC content of the clean data were calculated. All paired-end clean reads were aligned to a reference genome of *L. striatellus* using HISAT2 v2.0.5 (http://www.ccb.jhu.edu/software/hisat, accessed on 25 December 2018) and assembled by StringTie software [34] to generate mapped unigenes. The reference genome of *L. striatellus* (GigaDB RRID: SCR004002) and gene model annotation files were released by Zhu’s lab in the GigaScience repository [30]. All mapped unigenes were annotated by Gene Ontology (GO) enrichment analysis. GO terms with corrected *p*-value less than 0.05 were considered significantly enriched.

### 4.4. Analysis of Differentially Expressed Genes

Read counts mapped to each unigene were calculated by featureCounts v1.5.0-p3 [35]. FPKM of each unigene was obtained based on the length of the gene and reads count mapped to this gene. Unigene expression profiles were performed by heatmapping and Heml software [36]. DEGs between two treatments were identified using the edgeR R package [37]. Corrected *p*-value of 0.05 and absolute fold change of 2 were set as the threshold for significantly differential expression according to Benjamini and Hochberg method [38].

### 4.5. Identification and Comparative Expression Profiles of Olfactory Related Genes

Based on GO term described as odorant-binding/olfactory receptor activity, all unigenes were manually retrieved by keywords (OBP, CSP, OR, IR. GR SNMP, chemosensory protein, olfactory protein). Then, retrieved olfactory-related genes were matched with 128 olfactory genes in previous description [27] and identified using a BLASTx algorithm-based search in NCBI website. Expression profiles of these olfactory-related genes were displayed using Heml software according to FPKM of each gene [36].

### 4.6. Quantitative Real-Time PCR Analysis

Total RNA was isolated from 600 antennae, 100 legs and 50 heads of viruliferous and nonviruliferous adults using the TRIzol Total RNA Isolation Kit (Takara, Dalian, China). First-strand cDNA was synthesized using One Step SYBR PrimeScript RT-PCR kit (Takara, Dalian, China). Primers for *LstrOBPs* and *LstrActin* (control) (Table S2) were designed as previous description [27]. The qPCR was conducted with SYBR Premix Ex Taq (Takara) and a CFX96™ Real-Time PCR Detection System (Bio-Rad, Hercules, CA, USA) as follows: denaturation for 3 min at 95 °C, followed by 40 cycles at 95 °C for 10 s, and 60 °C for 30 s. Relative expression levels for triplicate samples were calculated using the 2^−ΔΔCt^ method [39], and expression levels of target genes were normalized to the *LstrActin* gene. Three technical repeats were performed for each of the three biological replicates. The *T*-test method was used to analyze the significance of relative expression level from various samples [40].

## 5. Conclusions

In summary, our transcriptomic analysis revealed the expression profiles of genes in antennae, legs and heads of *L. striatellus* infected by RSV. RSV infection regulated the expression of multiple olfactory-related genes in the a chemoreception organs. In particular, qPCR confirmed the down-regulation of *LstrOBP2*, and increased *LstrOBP3* and *LstrOBP9* in the chemoreception organs of females *L. striatellus* post RSV infection. It is suggested that RSV may alter host preference of the insect vector by changing these olfactory-related genes, which are ultimately beneficial to its transmission. Therefore, it is promising to develop approaches (such as RNAi based gene-silencing technology) to interrupt the olfactory-related genes in chemoreception organs of *L. striatellus* to control the transmission and spread of RSV in the future. In this regard, this work is a starting point for research, which will eventually help the control of RSV and provide new strategies for the control of other vector-borne viruses.

## Data Availability

Illumina sequencing reads from these transcriptomes were submitted to the NCBI Sequence Read Archive under accession number PRJNA589749.

## Supplementary Materials

The following are available online at https://www.mdpi.com/1422-0067/22/19/10299/s1, Figure S1: GO analysis of DEGs among male organs (or between female and male viruliferous organs), Table S1. Summary statistics of Illumina sequence data, Table S2: The primers used in this study.

## Abbreviations

DEGs	Differentially expressed genes
RSV	Rice stripe virus
CMV	Cucumber mosaic virus
PLV	Potato leafroll virus
TYLCV	Tomato yellow leaf curl virus
SRBDV	Southern rice black streaked dwarf virus
TCV	Tomato chlorosis virus
OBPs	Odorant-binding proteins
CSPs	Chemosensory proteins
IRs	Ionotropic receptors
GRs	Gustatory receptors
ORs	Odorant receptors
ORco	Olfactory co-receptor receptor
SNMPs	Sensory neuron membrane proteins
VFA	Viruliferous female antennae
VMA	Viruliferous male antennae
VFL	Viruliferous female legs
VML	Viruliferous male legs
VFH	Viruliferous female heads
VMH	Viruliferous male heads
NFA	Nonviruliferous female antennae
NMA	Nonviruliferous male antennae
NFL	Nonviruliferous female legs
NML	Nonviruliferous male legs
NFH	Nonviruliferous female heads
NMH	Nonviruliferous male heads
FPKM	Fragments per Kilobase of transcript per Million mapped reads
PCA	Principal component analysis
GO	Gene ontology
qPCR	Real-Time Quantitative PCR
VOC	volatile organic compounds
GC–EAD	gas chromatography–electroantennographic detection

## References

[1] Ng J.C.K., Falk B.W. (2006). Virus-Vector Interactions Mediating Nonpersistent and Semipersistent Transmission of Plant Viruses. Annu. Rev. Phytopathol..

[2] Hogenhout S.A., Ammar E.-D., Whitfield A.E., Redinbaugh M.G. (2008). Insect vector interactions with persistently transmitted viruses. Annu. Rev. Phytopathol..

[3] Mauck K.E., De Moraes C.M., Mescher M.C. (2010). Deceptive chemical signals induced by a plant virus attract insect vectors to inferior hosts. Proc. Natl. Acad. Sci. USA.

[4] Keesey I.W., Koerte S., Khallaf M.A., Retzke T., Guillou A., Grosse-Wilde E., Buchon N., Knaden M., Hansson B.S. (2017). Pathogenic bacteria enhance dispersal through alteration of Drosophila social communication. Nat. Commun..

[5] Ngumbi E., Eigenbrode S.D., Bosque-Pérez N.A., Ding H., Rodriguez A. (2007). Myzus persicae is arrested more by blends than by individual compounds elevated in headspace of plrv-infected potato. J. Chem. Ecol..

[6] Wang S., Guo H., Ge F., Sun Y. (2020). Apoptotic neurodegeneration in whitefly promotes the spread of tylcv. Elife.

[7] Hu K., Yang H., Liu S., He H., Li Y. (2019). Odorant-Binding Protein 2 is Involved in the Preference of Sogatella furcifera (Hemiptera: Delphacidae) for Rice Plants Infected with the Southern Rice Black-Streaked Dwarf Virus. Florida Entomol..

[8] Shi X.B., Wang X.Z., Zhang D.Y., Zhang Z.H., Zhang Z., Cheng J., Zheng L.M., Zhou X.G., Tan X.Q., Liu Y. (2019). Silencing of odorant-binding protein gene OBP3 using RNA interference reduced virus transmission of tomato chlorosis virus. Int. J. Mol. Sci..

[9] Renou M., Guerrero A. (2000). Insect Parapheromones in Olfaction Research and Semiochemical-Based Pest Control Strategies. Annu. Rev. Entomol..

[10] Morita H. (1972). Primary processes of insect chemoreception. Adv. Biophys..

[11] Benton R., Sachse S., Michnick S.W., Vosshall L.B. (2006). Atypical membrane topology and heteromeric function of Drosophila odorant receptors in vivo. PLoS Biol..

[12] Benton R., Vannice K.S., Vosshall L.B. (2007). An essential role for a CD36-related receptor in pheromone detection in Drosophila. Nature.

[13] Clyne P.J., Warr C.G., Carlson J.R. (2000). Candidate taste receptors in Drosophila. Science.

[14] Leal S.W. (2013). Odorant Reception in Insects: Roles of Receptors, Binding Proteins, and Degrading Enzymes. Annu. Rev. Entomol..

[15] Pelosi P., Iovinella I., Zhu J., Wang G., Dani F.R. (2018). Beyond chemoreception: Diverse tasks of soluble olfactory proteins in insects. Biol. Rev..

[16] Pelosi P., Zhou J.-J., Ban L.P., Calvello M. (2006). Soluble proteins in insect chemical communication. Cell. Mol. Life Sci. C.

[17] Pelosi P., Iovinella I., Felicioli A., Dani F.R. (2014). Soluble proteins of chemical communication: An overview across arthropods. Front. Physiol..

[18] Toriyama S. (1986). Rice stripe virus: Prototype of a new group of viruses that replicate in plants and insects. Microbiol. Sci..

[19] Falk B.W., Tsai J.H. (1998). Biology and molecular biology of viruses in the genus Tenuivirus. Annu. Rev. Phytopathol..

[20] Hibino H. (1996). Biology and Epidemiology of Rice Viruses. Annu. Rev. Phytopathol..

[21] Heong K.L., Cheng J., Escalada M.M. (2015). Rice Planthoppers—Ecology, Management, Socio Economics and Policy.

[22] Li Y., Chen D., Hu J., Zhang K., Liu F. (2020). The α-tubulin of Laodelphax striatellus mediates the passage of rice stripe virus (RSV) and enhances horizontal transmission. PLoS Pathog..

[23] Zhang F., Guo H., Zheng H., Zhou T., Zhou Y., Wang S., Fang R., Qian W., Chen X. (2010). Massively parallel pyrosequencing-based transcriptome analyses of small brown planthopper (Laodelphax striatellus), a vector insect transmitting rice stripe virus (RSV). BMC Genom..

[24] Yang M., Xu Z., Zhao W., Liu Q., Li Q., Lu L., Liu R., Zhang X., Cui F. (2018). Rice stripe virus-derived siRNAs play different regulatory roles in rice and in the insect vector Laodelphax striatellus. BMC Plant Biol..

[25] Lee J.H., Choi J.Y., Tao X.Y., Kim J.S., Kim W., Je Y.H. (2013). Transcriptome analysis of the small brown planthopper, Laodelphax striatellus carrying Rice stripe virus. Plant Pathol. J..

[26] Zhao W., Lu L., Yang P., Cui N., Kang L., Cui F. (2016). Organ-specific transcriptome response of the small brown planthopper toward rice stripe virus. Insect Biochem. Mol. Biol..

[27] Li Y., Hu J., Xiang Y., Zhang Y., Liu F. (2019). Identification and comparative expression profiles of chemosensory genes in major chemoreception organs of a notorious pests, Laodelphax striatellus. Comp. Biochem. Physiol. Part D Genom. Proteom..

[28] Li S., Zhou C., Zhou Y. (2019). Olfactory co-receptor Orco stimulated by Rice stripe virus is essential for host seeking behavior in small brown planthopper. Pest Manag. Sci..

[29] Xu Y., Fu S., Tao X., Zhou X. (2021). Rice stripe virus: Exploring Molecular Weapons in the Arsenal of a Negative-Sense RNA Virus. Annu. Rev. Phytopathol..

[30] Zhu J., Jiang F., Wang X., Yang P., Bao Y., Zhao W., Wang W., Lu H., Wang Q., Cui N. (2017). Genome sequence of the small brown planthopper, *Laodelphax Striatellus*. Gigascience.

[31] Naters W., Carlson J.R. (2006). Insects as chemosensors of humans and crops. Nature.

[32] Sim S., Ramirez J.L., Dimopoulos G. (2012). Dengue virus infection of the aedes aegypti salivary gland and chemosensory apparatus induces genes that modulate infection and blood-feeding behavior. PLoS Pathog..

[33] Wang G.Z., Zhou Y.J., Chen Z.X., University Z. (2004). Hangzhou Production of monoclonal antibodies to Rice stripe virus and application in virus detection. Acta Phytopathol. Sin..

[34] Pertea M., Kim D., Pertea G.M., Leek J.T., Salzberg S.L. (2016). Transcript-level expression analysis of RNA-seq experiments with HISAT, StringTie and Ballgown. Nat. Protoc..

[35] Yang L., Smyth G.K., Wei S. (2014). FeatureCounts: An efficient general purpose program for assigning sequence reads to genomic features. Bioinformatics.

[36] Deng W., Wang Y., Liu Z., Cheng H., Xue Y. (2014). HemI: A Toolkit for Illustrating Heatmaps. PLoS ONE.

[37] Robinson M.D., McCarthy D.J., Smyth G.K. (2010). EdgeR: A Bioconductor package for differential expression analysis of digital gene expression data. Bioinformatics.

[38] Haynes W., Dubitzky W., Wolkenhauer O., Cho K.H., Yokota H. (2013). Benjamini—Hochberg Method BT. Encyclopedia of Systems Biology.

[39] Rao X., Huang X., Zhou Z., Lin X. (2013). An improvement of the 2^−ΔΔCT^ method for quantitative real-time polymerase chain reaction data analysis. Biostat. Bioinform. Biomath..

[40] Livak K.J., Schmittgen T.D. (2001). Analysis of relative gene expression data using real-time quantitative PCR and the 2^−ΔΔCT^ method. Methods.

