# Supplementary figures and images for: Comparative Transcriptome Analysis of Chemoreception Organs of Laodelphax striatellus in Response to Rice Stripe Virus Infection
Source: Int J Mol Sci. 2021 Sep 24;22(19):10299. doi: 10.3390/ijms221910299 (PMC8532003; doi:10.3390/ijms221910299)

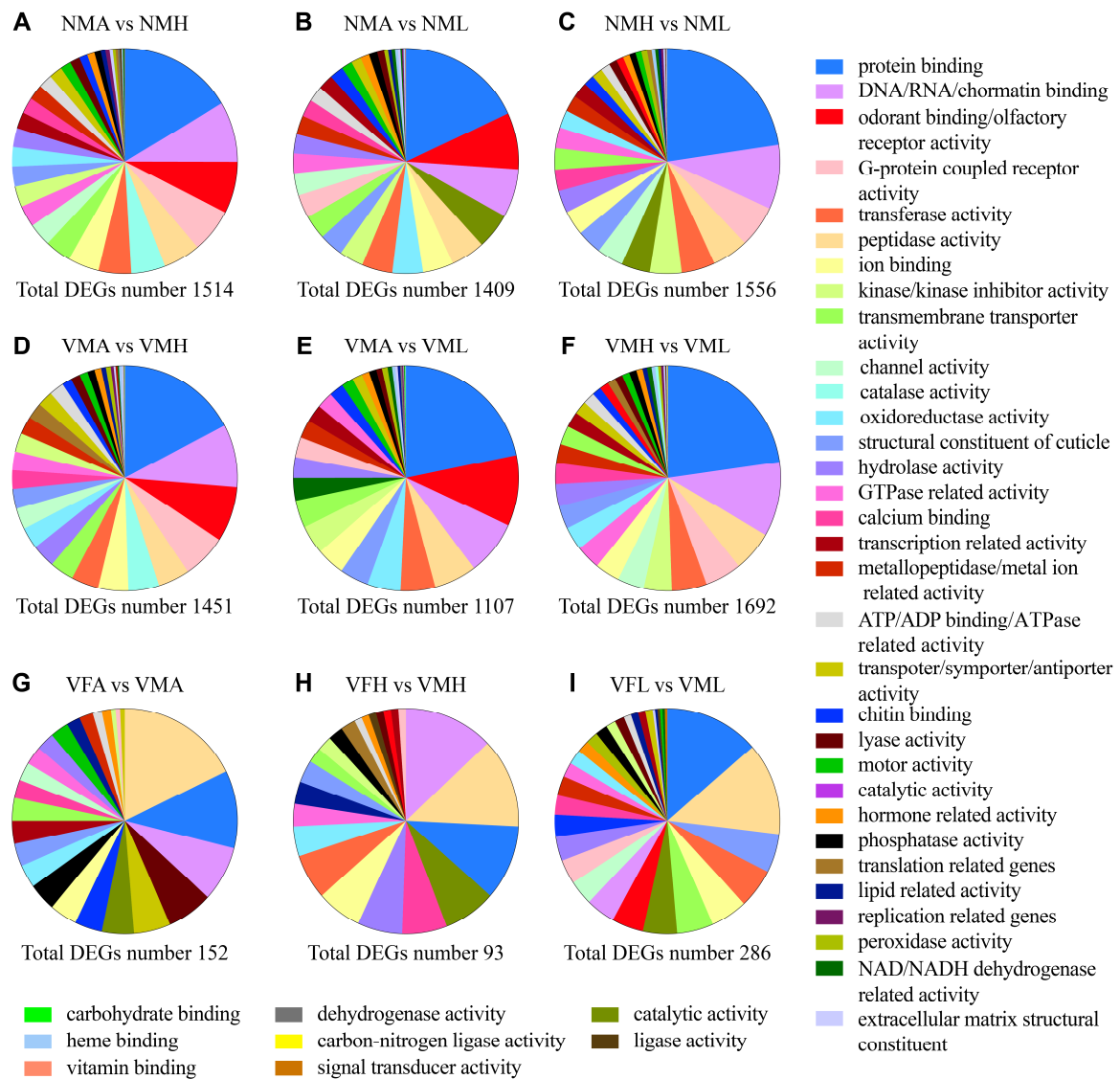

**Figure S1.** GO analysis of DEGs among male organs (or between female and male viruliferous organs).

Supplement: Supplementary file 1 [file ijms-22-10299-s001.zip › Figure S1.pdf]
